# Advancement of regulating cellular signaling pathways in NSCLC target therapy via nanodrug

**DOI:** 10.3389/fchem.2023.1251986

**Published:** 2023-09-07

**Authors:** Wenqiang Li, Mei Li, Qian Huang, Xiaoyu He, Chen Shen, Xiaoming Hou, Fulai Xue, Zhiping Deng, Yao Luo

**Affiliations:** ^1^ Zigong First People’s Hospital, Zigong, Sichuan, China; ^2^ West China Hospital, Sichuan University, Chengdu, Sichuan, China; ^3^ Sichuan North Medical College, Nanchong, Sichuan, China

**Keywords:** nanodrug, signaling pathways, non-small cell lung cancer, drug resistance, targeted therapy

## Abstract

Lung cancer (LC) is one of the leading causes of high cancer-associated mortality worldwide. Non-small cell lung cancer (NSCLC) is the most common type of LC. The mechanisms of NSCLC evolution involve the alterations of multiple complex signaling pathways. Even with advances in biological understanding, early diagnosis, therapy, and mechanisms of drug resistance, many dilemmas still need to face in NSCLC treatments. However, many efforts have been made to explore the pathological changes of tumor cells based on specific molecular signals for drug therapy and targeted delivery. Nano-delivery has great potential in the diagnosis and treatment of tumors. In recent years, many studies have focused on different combinations of drugs and nanoparticles (NPs) to constitute nano-based drug delivery systems (NDDS), which deliver drugs regulating specific molecular signaling pathways in tumor cells, and most of them have positive implications. This review summarized the recent advances of therapeutic targets discovered in signaling pathways in NSCLC as well as the related NDDS, and presented the future prospects and challenges.

## 1 Introduction

Cancer is the main cause of death worldwide, and lung cancer (LC) is the disease with the highest mortality rate (Ferlay et al., 2018; Ferlay et al., 2019). According to histology, LC is generally divided into two categories: small cell lung cancer (SCLC) and non-small cell lung cancer (NSCLC). Here, NSCLC is the most common type of LC (85%), with high incidence rate and high mortality (Ferlay et al., 2019). Moreover, the average survival rate of patients is 10%–20% (Ferlay et al., 2018). Nowadays, targeted drugs for gene mutations and immune loci have become the preferred option for NSCLC patients, but the current clinical route of drug delivery still has major drawbacks, such as low drug utilization and significant side effects, which reduce the survival benefits for clinical patients (Hirsch et al., 2017; de Scordilli et al., 2022). In addition, there are many problems inherent in the traditional route of drug delivery. The bioavailability of oral and intravenous dosing is low, and these systemic dosing regimens can cause a variety of toxic reactions in the body, including severe vomiting, seizures, vasculitis, and even death, clearly doing more harm than good in low-risk stage IA NSCLC (NSCLC Meta-analysis Collabora tive Group, 2014; Argilés et al., 2023). Therefore, developing new therapeutic interventions which focus on more microscopic and detailed levels like the signaling pathways of disease onset to overcome these limitations is of great significance. Scientists have done a great deal of work in exploring the evolution of tumor cells based on specific molecular signals for drug treatment and targeted delivery. Targeted therapy has become a hot term in tumor treatment with a broad meaning that includes not only the targeting of drugs but also the precise delivery of drugs.

For the proliferation and invasion process of LC cells, abnormal cell signaling pathways, which exist complex regulatory mechanisms, are closely related to genetic mutations. Mutations of *RAS* gene are common in NSCLC, and most of them are *KRAS* mutations (Punekar et al., 2022). Mitogen-activated protein kinase (MAPK) is one of the major signals stimulated by *RAS* (Ferlay et al., 2018; Ferlay et al., 2019). The *RAS* rapid fibrosarcoma (RAF)-MAPK-extracellular signal-regulated kinase (ERK) pathway and phosphatidylinositol 3-kinase (PI3K)-protein kinase B (AKT) pathway control cell survival and proliferation in NSCLC (Ferlay et al., 2019). *Epidermal growth factor receptor (EGFR)* gene mutations are important in the development of NSCLC. It can increase EGFR expression when mutations in kinase, resulting in the functional upregulation of the EGFR pathway and uncontrolled proliferation of mutant tumor cells (Passaro et al., 2021). In malignant cells, vascular endothelial growth factor (VEGF) and VEGF receptor (VEGFR) promote cell proliferation, survival, and angiogenesis in, and inhibition of VEGF and VEGFR retards tumor growth (Niu et al., 2022). Also important in cell differentiation, proliferation, and cancer progression are the pathways NTRK/ROS1 and JAK-STAT (Lai and Johnson, 2010; Song et al., 2023). In summary, molecular signaling has a strong connection with tumor progression, which is also can modulate drug resistance. To fully understand the functions of these pathways, it is necessary to examine their upstream and downstream node. As evidence accumulates, strategies that target these pathways may hold promise for NSCLC treatment. However, the complexity of signaling makes it difficult to understand the complete regulatory pathway of a particular signaling target in tumor cells. At the same time, all mutated genes may become therapeutic targets, while there are still many unknown mutated genes, so that continuing in-depth screening of mutated genes has a positive effect on drug development.

Accompanied by the discovery of more and more signaling pathways, a great victory has been achieved in the precise molecular therapy of NSCLC, which established in regulating signaling pathways to overcome the drug resistance. For example, activation of NF-κB/Bcl-2/Snail pathway increases chemotherapy resistance in NSCLC, and thus targeted drug delivery of this pathway would behave with good specificity and pharmacokinetic characteristics which could inhibit tumor cell proliferation (McCubrey et al., 2008; Chen et al., 2015; Asati et al., 2016). Targeted drugs can reduce resistance to chemotherapy drugs, but are prone to cause new genetic mutations during treatment, leading to resistance to targeted drugs (Cabanos and Hata, 2021). Targeting EGFR tyrosine kinase inhibitors (EGFR-TKIs) for NSCLC promotes the emergence of acquired drug resistance, a major barrier to EGFR-targeted therapy (Johnson et al., 2022). EGFR resistance mechanisms are mainly classified as dependent resistance and non-dependent resistance. Dependent resistance mechanisms include T790M mutation, C797 mutation and G796R mutation, while non-dependent resistance mechanisms are divided into mesenchymal-epithelial transition (MET) factor amplification, (human epidermal growth factor receptor 2) HER2 amplification and gene fusion (Passaro et al., 2021; Nie et al., 2022). Apart from that, the PI3K/AKT/mTOR signaling pathway can activate EGFR and mutate among various malignant tumors, including leukemia and NSCLC (Yan et al., 2021; Chen et al., 2022). Studies have confirmed that the knockdown of *miR-126*, *miR-203*, and *miR-34a* genes can regulate drug resistance through PI3K/AKT signaling pathway (Zhong et al., 2010; Garofalo et al., 2011). It has to be acknowledged that some unknown signaling pathways may have direct or indirect effects of the drug resistance.

In addition to the drug resistance of cancer cells, the positive and negative role of the signaling molecules in the immune system in drug therapy is also being paid attention to. Immune-related regulators play key roles in autoimmunity, self-tolerance, and the malignant microenvironment, such as the co-suppressor receptor programmed death 1 (PD-1) and its ligand (PD-L1) (Reck et al., 2022). Furthermore, there is substantial evidence that cancer cells may use these immunomodulatory factors to evade the immune response (Muenst et al., 2015). PD-1/PD-L1 pathway immunologic drugs have been widely used in the treatment of NSCLC (Suresh et al., 2018; Reck et al., 2022). At the same time, immunotherapeutic drugs, when applied to the human body, are distributed to various tissues and organs throughout the body and cannot achieve precise focal targeting, thus causing a series of immune-related adverse reactions, which greatly hinders their clinical application potential (Jing et al., 2021; Zhou et al., 2023). To overcome these problems and improve patient outcomes, nanoparticles (NPs) with penetrating and slow release properties have been proposed to successfully treat drug-resistant cancer cells of NSCLC *in vitro* or *in vivo* models (Palazzolo et al., 2018; Patra et al., 2018; Wang et al., 2018). The results of these studies have stimulated the interest of researchers in nanomaterials, and more research is still in progress.

The emergence of drug resistance and apparent toxicity is currently an important reason for the unsustainable treatment of NSCLC patients. NPs are endowed with special functions due to their microstructure, which bring more hope to address these problems. In particular, the modified NPs have the advantage of targeting and carrying multiple drugs. Nanotechnology requires multidisciplinary cooperation, involving various types of NPs as well as new nanodevices and applications of nanomaterials in different fields (Molina et al., 2008; Sung et al., 2021). NPs have a wide surface area and can be modified by bonding or encapsulation (Rosell et al., 2020). As carriers of antineoplastic drugs, NPs have greatly improved efficacy, safety, stability and pharmacokinetics of drugs (Naylor et al., 2016; Stater et al., 2021; Haider et al., 2022; Tian et al., 2022; Detappe et al., 2023; Fang et al., 2023; Nguyen et al., 2023). The researches on tumor targeted therapy via NPs focus on the size and the impact of encapsulation techniques on the bioavailability of drugs *in vivo* (Raju et al., 2021; Ezhilarasan et al., 2022; Huang et al., 2022). Biocompatible nanocarriers can be tailored to tumor characteristics to improve their physical and chemical properties, permeability, and metabolism, such as the smaller structures can penetrate tissue barriers more easily (Seeta Rama Raju et al., 2015). Furthermore, researchers can limit the uptake of drugs in healthy tissues by wrapping appropriate NPs to enhance drug targeting, thereby shield normal cells from the cytotoxic effects of anticancer drugs, and reduce adverse drug reaction (Kalyane et al., 2019; Haider et al., 2020; Duan et al., 2022; Haider et al., 2022).

**FIGURE 1:**
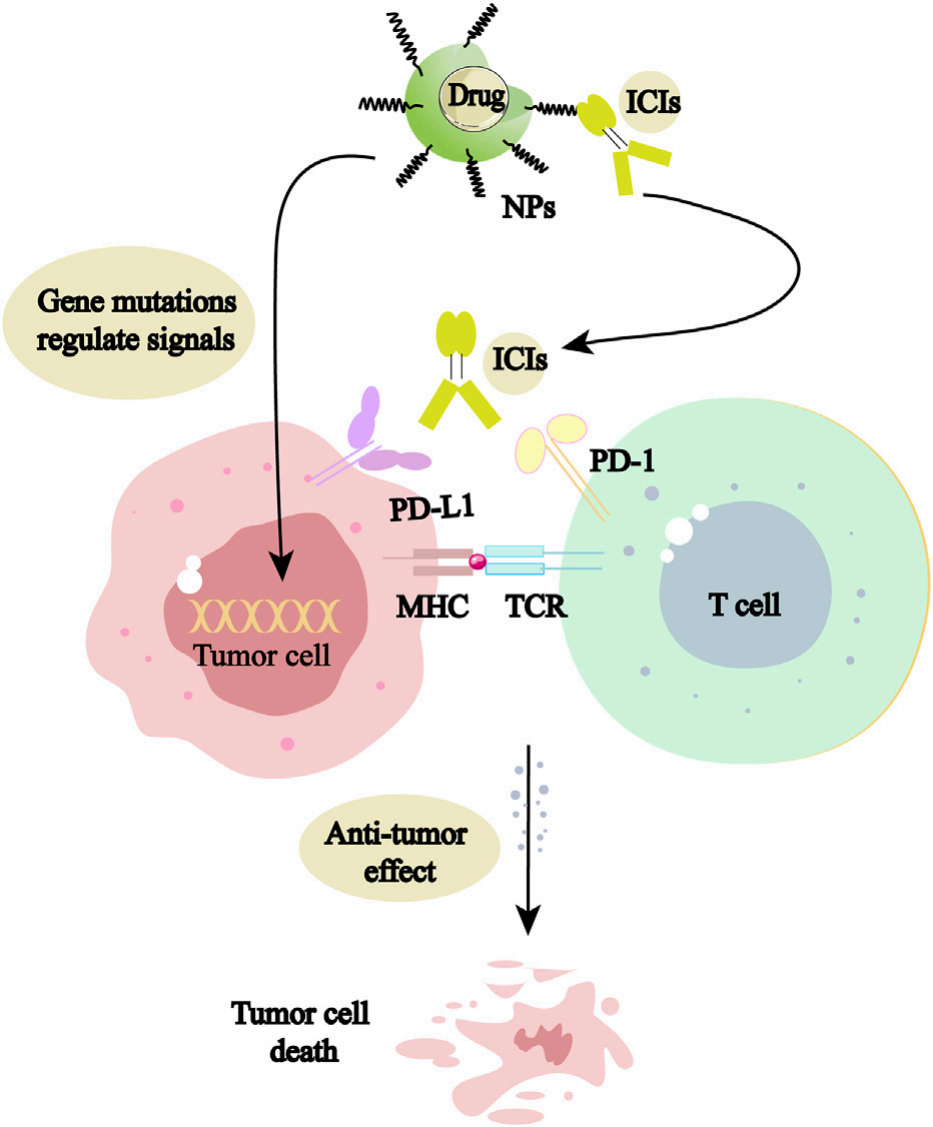
NPs can be used as delivery vehicles for mutation-targeting drugs and immune checkpoint inhibitors (ICIs), acting directly on tumor cells or activating living immune cells, respectively, to promote tumor cell death.

Nano-based drug delivery systems (NDDS) formed by NPs have become a hot issue for research. NDDS has a wide range of promising applications in NSCLC treatment and has the potential to save patients’ lives. Targeted therapy aiming at mutated genes and immune targets is of epoch-making significance in NSCLC treatment. So far, the abundant studies on signaling targets based on NDDS have performed well in the delivery of targeted and immune drugs, and the mechanism is shown in Figure 1. NDDS is a new therapeutic concept, which is theoretically well synergistic with the drug itself, providing benefits to patients, and makes the goal of long-term coexistence with malignancy the technical and theoretical basis. It will break through the limitations of traditional treatments and promotes NSCLC patients to live longer.

In this paper, we summarize the results achieved in recent years regarding the discovery of therapeutic targets in signaling pathways of NSCLC and related NDDS, and present the future prospects and challenges.

## 2 Strengths of nanodrug in NSCLC treatment

The pathogenesis of NSCLC is mediated by multiple intercellular molecular signaling pathways, and the targeted therapeutically relevant signaling targets are also receiving more attention. Gene-targeted therapy is widely used in the treatment of NSCLC, where EGFR classical mutations and KRAS G12C/D/V occur more frequently, while ROS1 rearrangements, RET rearrangements, NTRK fusions, MET14 exon skipping mutation, BRAF V600E mutations are relatively infrequent. They can be classified as “rare” mutations, but all of them have corresponding targeted drugs and should not be ignored (Harada et al., 2023). However, the physicochemical properties of these targeted drugs are always poor. Besides, the complex multi-order biological barriers in the body often lead to treatment that does not achieve the desired efficacy and also brings certain side effects on the organism (Boolell et al., 2015). More seriously, some side effects can even hasten the patient’s death.

In the immune system, T cell-mediated cellular immunity is the “main legion” for tumor eradication, where T cell activation is required to exert anti-tumor effects (Tanaka and Sakaguchi, 2017; Liu et al., 2020). But some “cunning” tumor cells can bypass immune surveillance and grow uncontrollably, thus endangering the health of the body. These tumor cells manipulate the immune cells by using the characteristics of immune checkpoints, and express PD-L1 protein on the surface, which causes T cells to lose the activity of killing tumor cells, thus realizing the immune escape (Jiang et al., 2019; Kim et al., 2019; Liu Z. et al., 2021). Immune checkpoint inhibitors (ICIs) can effectively overcome tumor immune evasion, and antagonists targeting immune checkpoint ligands can effectively activate tumor-specific T cells (Zhang et al., 2020; Liu et al., 2022). But traditional immune checkpoint blockade therapies have disadvantages, such as low immunogenicity, weak targeting, easy drug resistance, and accidental cytokine storms (Vincent et al., 2022). NDDS can effectively enhance ICIs’ blocking efficiency. At the same time, it can achieve synergistic tumor treatment in combination with other therapeutic tools (Vincent et al., 2022).

**FIGURE 2:**
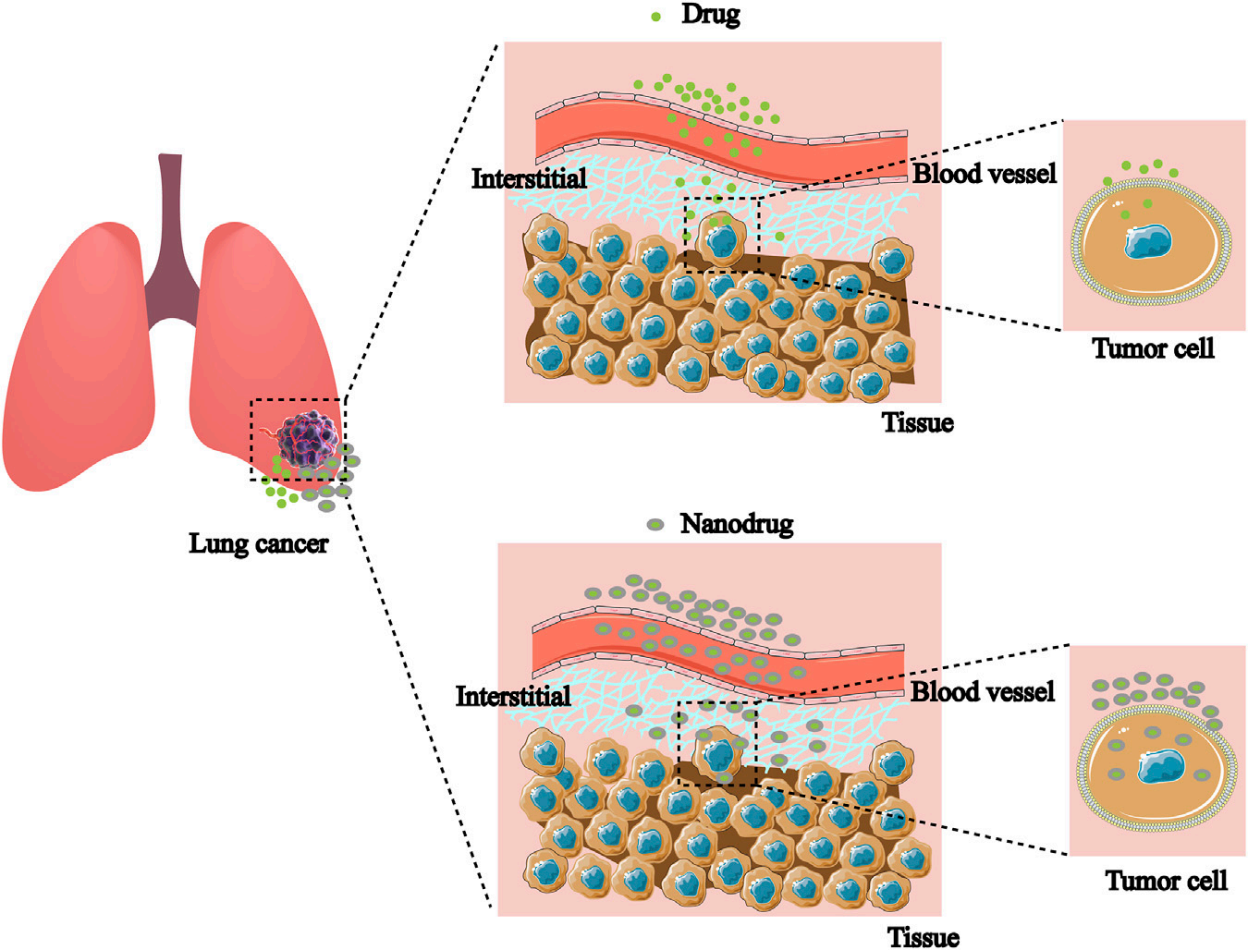
Nanodrugs are more likely to break through cellular and tissue barriers to accumulate in target cells than non-nanodrugs.

**FIGURE 3:**
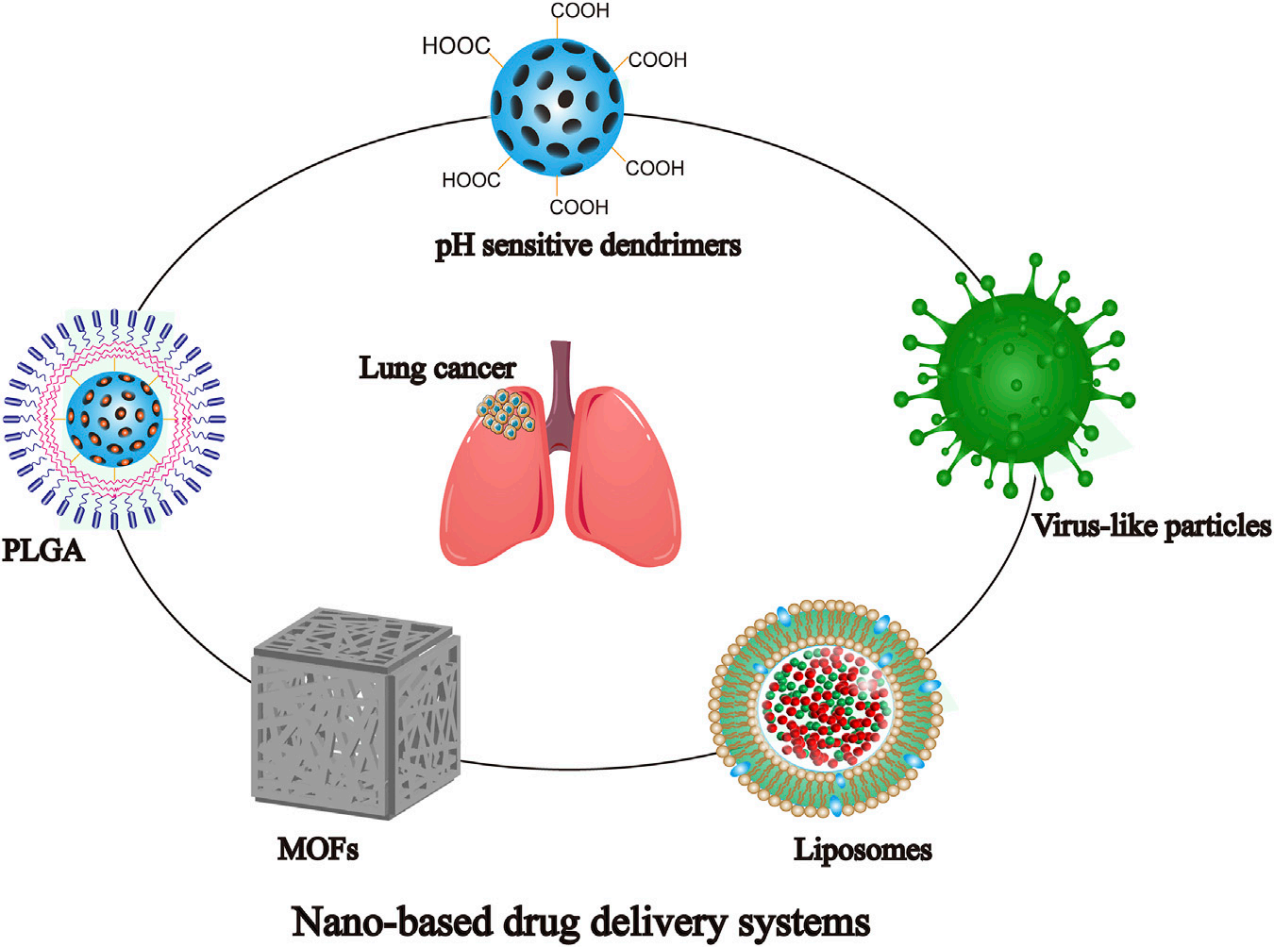
Different types of nanomaterials commonly used for NDDS.

NDDS can control the distribution and metabolic behavior at different levels of tissues, cells, and organelles by improving the stability and physicochemical properties of drugs, as well as by overcoming multi-level biological barriers (Chen et al., 2014; Wang J. et al., 2021). Similarly, it has significant implications for the regulation of drug resistance (Vincent et al., 2022). Based on the anatomical and pathophysiological differences between normal and tumor cells, the NPs have strong enhanced permeability and retention (EPR) effect, as shown in Figure 2 (Ikeda-Imafuku et al., 2022; Wu et al., 2023). Nanocarriers can be divided into natural polymer and synthetic polymer materials, most of which have excellent biocompatibility, stability, safety, non-toxicity and modifiability, mainly including virus-like particles, poly lactic glycolic acid (PLGA), liposomes, dendrimers, metal organic framework compounds, as shown in Figure 3 (Guo and Jiang, 2020; Ji et al., 2023). Meanwhile, many biomaterials are inherently targeted, so that nanomaterial delivery systems are always partnered with nucleic acid drugs (Zhang et al., 2013). Due to the emergence of drug resistance, targeting related signaling pathways based on nanomaterials has become a new direction to treat NSCLC in recent years, as shown in Table 1.

**TABLE 1:** Examples of targeted signaling pathways based on nanomaterials for the treatment of NSCLC.

Nanocarriers	Drugs	Mechanisms	Results	Ref
Magnetic lentivirus	RNAi	Microscale RNAi was achieved by EGFR silencing	Leading to EGFR-TKIs resistant H19750 cell apoptosis	Arrieta et al. (2012)
HA-6-(2-nitroimidazole) hexylamine-LOX	AAV2	Activation and release of lactate lead to specific delivery in tumor for virotherapy	Promoting cell apoptosis and inhibiting proliferation	Tseng et al. (2018)
PEGHis0.5-PEG-Glu0.5	—	Activating the p53 mediated apoptosis signaling pathway and inducing the production of light induced ROS in NSCLC cells	Suppressing cell growth and resensitizing cancer cells	Li et al. (2016)
RNA NPs	siRNA	Silencing anti-apoptotic factor Survivin	Chemotherapy sensitization and tumor regression	Li et al. (2021)
FITC-AEYLR	—	FITC-AEYLR has high EGFR targeting ability, enhancing cell specific uptake *in vitro*	—	Han et al. (2013)
apt-CL-E	erlotinib	Promoting the uptake of erlotinib in EGFR mutated cancer cells	Causing cell cycle arrest and apoptosis	Li et al. (2017)
PEG-PLA	CsA/Gef	CsA formed in NPs is inactivated through the STAT3/Bcl-2 signaling pathway	Sensitizing Gef resistant cells and drug-resistant tumors to Gef therapy	Han et al. (2018)
NP-DUAL	Gefitinib	Adjusting STAT3/miR-21/AKT/PTEN/HIF-1α axis to restore MET	Sensitizing drug-resistant cells again	Upadhyay et al. (2021)
PVI	siRNA	Silencing VEGF in cancer cells	Inhibiting cell proliferation and metastasis	Kandasamy et al. (2020)
QDs	—	Collaborative PTT/PDT	Inhibiting NSCLC without significant toxicity	Kuo et al. (2018)
QDs	NIR	Promoting the production of reactive oxygen species by regulating the PI3K/AKT pathway	Triggering cell death	Liu et al. (2021b)
Carboxymethyl chitosan	siRNA	Resulting in a significant decrease in STAT3 expression	Increasing cell apoptosis rate and inhibiting proliferation	Chen et al. (2020)
LNPs	siRNA	Silencing STAT3 and HIF-1α	Causing an increase in macrophage levels and increasing anti-tumor efficacy	Shobaki et al. (2020)
SLNs	RNAi	Downregulating STAT3	Sensitizing cancer cells resistant to cisplatin	Kotmakçı et al. (2017)
PLA	Erlotinib/fedratinib	Downregulating expression of p-EGFR, p-JAK2, p-STAT3, and Survivin in the JAK2/STAT3 signaling pathway	Reversing ELTN resistance and synergistic anti-cancer effect	Chen et al. (2018)
RBCm-OM/PLGA	OM	Reducing the expression level of Bcl-2, promoting the release of cell c in the cytoplasm, and activating Caspase-3	Reducing cancer cell proliferation *in vitro*, inducing apoptosis *in vivo*, and inhibiting tumor growth	Chen et al. (2020)
PVP-b-PCL	Tet	Up-regulating the expression of TIMP-3, Bcl-2 and Bcl-xl, down-regulating the expression of MMP2 and MMP9	Enhancing cell apoptosis, and inhibiting cell migration and invasion	Xu et al. (2014)
Cationic immunoliposo-me	Plasmid DNA	Downregulation of immunosuppressive molecules and addition of cytotoxic T cell activity	Restoring effective immune response to lung cancer cells	Kim et al. (2022)
Chlorin e6-encapsulated fluorinated dendrimer	CRISPR/Cas9	F-PC/pHCP under 660 nm laser activated the HSP70 promoter and enabled the specific expression of the Cas9 protein to disrupt the *PD-L1* gene, and prevent immune escape	Inducing immunogenic cell death of tumor cells, and inhibiting tumor growth	Zhao L. et al. (2022)
ZnPP@MSN	RGDyK	Integrin β3 (β3-int) is strongly upregulated in NSCLC-SM. Its inhibitor RGDyK promotes PD-L1 ubiquitination	Reversing the immune escape of cancer cells	Zhou et al. (2022)
Nanodiamond-dox	Orubicin	Induction of PD-L1 as well as NF-κB-dependent RAGE expression in tumor cells via the RAGE/NF-κB pathway enhances the role of HMGB1′S	Inhibiting tumor cell growth	Xu et al. (2021)

Abbreviations: Ref.: references; EGFR: epidermal growth factor receptor; EGFR-TKIs: EGFR, tyrosine kinase inhibitors; HA: hexylamine acid; LOX: lactate oxidase; AAV2: adeno-associated virus serotype 2; PEG: polyethylene glycol; His: histidine; Glu: glutamic acid; ROS: reactive oxygen species; NSCLC: non-small cell lung cancer; NPs: nanoparticles; siRNA: small interfering RNA; FITC: fluorescein isothiocyanate; Apt-CL-E: Apt-Cs-anchored liposomal complexes; PLA: poly (D,L-lactic acid); CsA: cyclosporin A; Gef: Gefitinib; NP-DUAL: transferrin modified poly lactic glycolic acid thymidine nanoparticle combined with gefitinib; MET: mesenchymal-epithelial transition factor; PVI: poly (1-vinylimidazole); QDs: quantum dots; PTT: photothermal therapy; PDT: photodynamic therapy; NIR:Near-infrared; LNPs: lipid nanoparticles; SLNs: solid lipid nanoparticles; ELTN: erlotinib; RBCm-OM/PLGA: combination of polylactic acid glycolic acid (PLGA) and red blood cell membrane (RBCm) to wrap obatok mesylate (OM); PVP-b-PCL: poly (N-vinylpyrrolidone) block poly (ε-Caprolactone); Tet: tetrandrine; CRISPR: clustered regularly interspaced short palindromic repeats; PD-L1: programmed cell death 1 ligand 1; ZnPP: zinc protoporphyrin; MSN: mesoporous silica nanoparticles; RAGE: receptor for advanced glycation endproducts; TAMs: tumor-associated macrophages.

## 3 Nanodrug targeted epidermal growth factor receptor in NSCLC

The EGFR is one of the most common mutation driver oncogenes. Among Asian female nonsmokers with NSCLC, the mutation rate was as high as 59.4%, with exon 19 deletions and L858R point mutations located in the receptor tyrosine kinase (RTK) domain accounting for 90% of mutations (Li et al., 2016; Bílek et al., 2019). The stimulation of MAPK and PI3K is highly associated with increased cancer risk, and the downstream signaling pathways are associated with cell proliferation, metastasis, and drug resistance (Villaruz et al., 2013; Ciuffreda et al., 2014). Drugs targeting the EGFR have been developed rapidly, such as gefitinib, afatinib, and osimertinib (Yang et al., 2020; Lai-Kwon et al., 2021; Li et al., 2022; Liam et al., 2023). Despite the significant effect of initial treatment, patients often develop acquired resistance after a period of time, by mechanisms including dependence or non-dependence on the EGFR pathway (Taniguchi et al., 2019; Chmielecki et al., 2023). To solve these problems, NPs with special properties such as slow release have been shown initial success (Huang et al., 2022).

The latest monotherapies to address resistance to EGFR-TKIs mainly include nanoconjugates viral delivery, nucleic acid therapy, and targeted EGFR-TKIs loaded in nanostructures. Viruses as natural carrier materials take advantage of the biochemical and physical properties, such as solubility and nanometer size, making them an important choice for NDDS (Sainsbury and Steinmetz, 2023). Arrieta’s team constructed a novel type of magnetic lentivirus that can infect EGFR-TKIs-resistant cells in the model and realize microscale RNA interference (RNAi) by inhibiting EGFR expression, causing apoptosis of drug-resistant cells (Song et al., 2009; Li et al., 2021). In addition, pH affects the effect of nanocarriers mainly by microenvironment/nanomaterial surface charge interconversion, tumor penetration size, and swelling or disintegration upon drug release (Song et al., 2008). The lactate accumulation method was used to design lactate-responsive vectors containing lactate oxidase (LOX) and AAV2, which reduced pH and viral infection, as well as increased apoptosis when both LOX and lactate were presented in the formulation (Tseng et al., 2018).

Nanocarriers can carry nucleic acids through the phospholipid bilayer of cell membranes, which promotes easier drug accumulation within the target cells (Bishop et al., 2015). Li et al. reported that exosomes loaded with small interfering RNA (siRNA) were used for the suppression of NSCLC (Li et al., 2021). Cholesterol is used to anchor ligands targeting EGFR onto secretions that load siRNA to silence the anti-apoptotic factor survivin. Cytoplasmic delivery of siRNA resolves the problem of endosomal capture and leads to effective gene knockdown, chemosensitization and tumor regression. In NSCLC patients, the knockdown of expression of selected appropriate targets restores sensitivity to EGFR-targeted drugs (Li et al., 2021). Thereby, the progress has been made in combining RNA nanotechnology with exon-delivery platforms, which can improve the targeting of cancer therapies.

The discovery of ligands that bind specifically to cancer cells is essential for NDDS delivery, and peptide binding to liposomes has been used to selectively deliver drugs to kill tumor cells with EGFR mutations (Song et al., 2008; Song et al., 2009). The study was performed by small peptides with phosphorylation sites (AEYLR, EYINQ, and PDYQQD), which were labeled with fluorescein isothiocyanate (FITC), in tumor cells of NSCLC with or without EGFR expression. It showed that AEYLR recognizes EGFR protein with high selectivity (Han et al., 2013). This confirmed that NDDS bound to AEYLP can accumulate more easily in tumor cells. Li et al. prepared lipid-containingNPs loaded with anti-EGFR DNA aptamer, which can make NDDS reach the target cells more easily by exploiting the specific binding of EGFR DNA aptamer, thus increasing the drug concentration and exerting better anti-tumor efficacy of targeted drugs (Li et al., 2017). Additionally, Han et al. loaded cyclosporine A and targeted drugs in a nanostructured poly (ethylene glycol)-poly (lactic acid) (PEG-PLA) and found a significant effect of cyclosporine A in reversing resistance to EGFR-TKIs (Han et al., 2018). Compared to EGFR-TKIs alone, NPs loaded with EGFR TKI not only reduce systemic toxicity but also improve intracellular delivery and increase bioavailability (Ahlawat et al., 2022). Upadhyay et al. demonstrated the efficiency of transferrin-modified PLGA thymoquinone NPs combined with gefitinib (NP-DUAL-3) in treating resistant NSCLC cells for the first time (Upadhyay et al., 2021). The results indicated that NP-DUAL-3 may restore the MET phenomenon, thereby making drug-resistant NSCLC cells re-sensitive to gefitinib. Therefore, the combination of NPs and gefitinib may be effective in treating NSCLC patients in the future.

## 4 Nanodrug targeted vascular endothelial growth factor receptor in NSCLC

VEGF is mainly secreted by vascular endothelial cells, as well as tumor microenvironment (TME) cells, such as tumor-associated macrophages (TAMs) (Hwang et al., 2020), tumor-associated neutrophils (TANs) (Guimarães-Bastos et al., 2022), mast cells (MCs) (Komi et al., 2020), myelogenous suppressor cells (MDSCs) (Mortezaee, 2021) and natural killer cells (NKs) (Eisinger et al., 2020). VEGF is the main mediator of tumor microangiogenesis and closely associated with the development and progression of NSCLC. In addition, VEGF stimulates regulatory immune cells by inhibiting antigen presentation, thus promoting immunosuppression of the TME. It is an important manifestation of VEGF’s involvement in immune regulation (Zhao Y. et al., 2022).

The binding of VEGF ligands to VEGFR-2 and the PI3K/AKT signaling pathway control the survival of endothelial cells (Wang et al., 2022). The activation of endothelial nitrogen monoxide (NO) synthase by c-Src and phospholipase C1 (PLC1), and the activation of prostacyclin synthase by Raf1-MEK1/2ERK1/2 lead to an increase of NO and PGI2 in endothelial cells, respectively (Socinski et al., 2018). This pathway is the core of endothelial cell proliferation. The upregulation of NO induced by VEGF may also participate in the generation and mobilization of endothelial progenitor cells (Aicher et al., 2003).

Kandasamy et al. explored the efficiency of poly (1-vinyl imidazole) (PVI) as an effective siRNA carrier for *VEGF* gene silencing (Kandasamy et al., 2020). They found that the individual PVI polymer was safe to the cells, and the polymer exhibited good internalization and effectively escaped the inner body, indicating that the vector may be a biocompatible system for gene therapy. In terms of silencing *VEGF* in tumors, the polymerase is more effective than free siRNA, and the silencing of *VEGF* leads to changes in the gene expression responsible for cancer cell proliferation and metastasis. Peptide silencing of *VEGF* can enhance the cytotoxicity of chemotherapy drug 5-fluorouracil, suggesting that it could be used as an adjuvant treatment strategy for cancer. Meanwhile, VEGF-targeted RNAi using poly-siRNA/tGC NPs in combination with chemotherapeutic agents can control tumor growth by increasing anti-angiogenic efficacy while minimizing toxicity and drug resistance (Kwak et al., 2017). Chemically polymerized siRNAs complexed with thiolated-glycol chitosan (psi (VEGF)/tGC) NPs mediated suppression of VEGF which exerted anti-tumor effects. Furthermore, the combination of bevacizumab can better perform the drug’s efficacy (Kim et al., 2017).

## 5 Nanodrug regulated PI3K/AKT/mTOR signaling pathway in NSCLC

The PI3K/AKT/mTOR pathway is vital in regulating cell growth and metabolism, which is significantly activated in NSCLC (50%–73%) (Papadimitrakopoulou, 2012). Meanwhile, persistent activation of this pathway can contribute to the development of resistance to anticancer therapy. PI3K is regulated by numerous upstream factors, such as HER2 (Tan, 2020). Under stress or ligand binding, AKT is readily activated to regulate the phosphorylation of phosphatidylinositol 4,5-bisphosphate (PIP2) to phosphatidylinositol 3,4,5-trisphosphate (PIP3) (Papadimitrakopoulou, 2012). Activation of AKT will cause changes in downstream signaling molecules, which can inhibit Bcl-2-associated death promoter (BAD) and Bcl-2-associated X protein (BAX), members of the Bcl-2 family, and promote apoptosis (Cantley, 2002). Activation of nuclear factor-κB (NF-κB) light chain enhancer plays a role in immune regulation and biological behavior (Sonenshein, 1997). Another important downstream pathway is the activation of protein kinase mTOR. The mTOR can cause activation of the eukaryotic translation initiation factor 4 complex, which subsequently promotes tumor development, regulates cell cycle, and inhibits cell apoptosis (Engelman et al., 2006).

**FIGURE 4:**
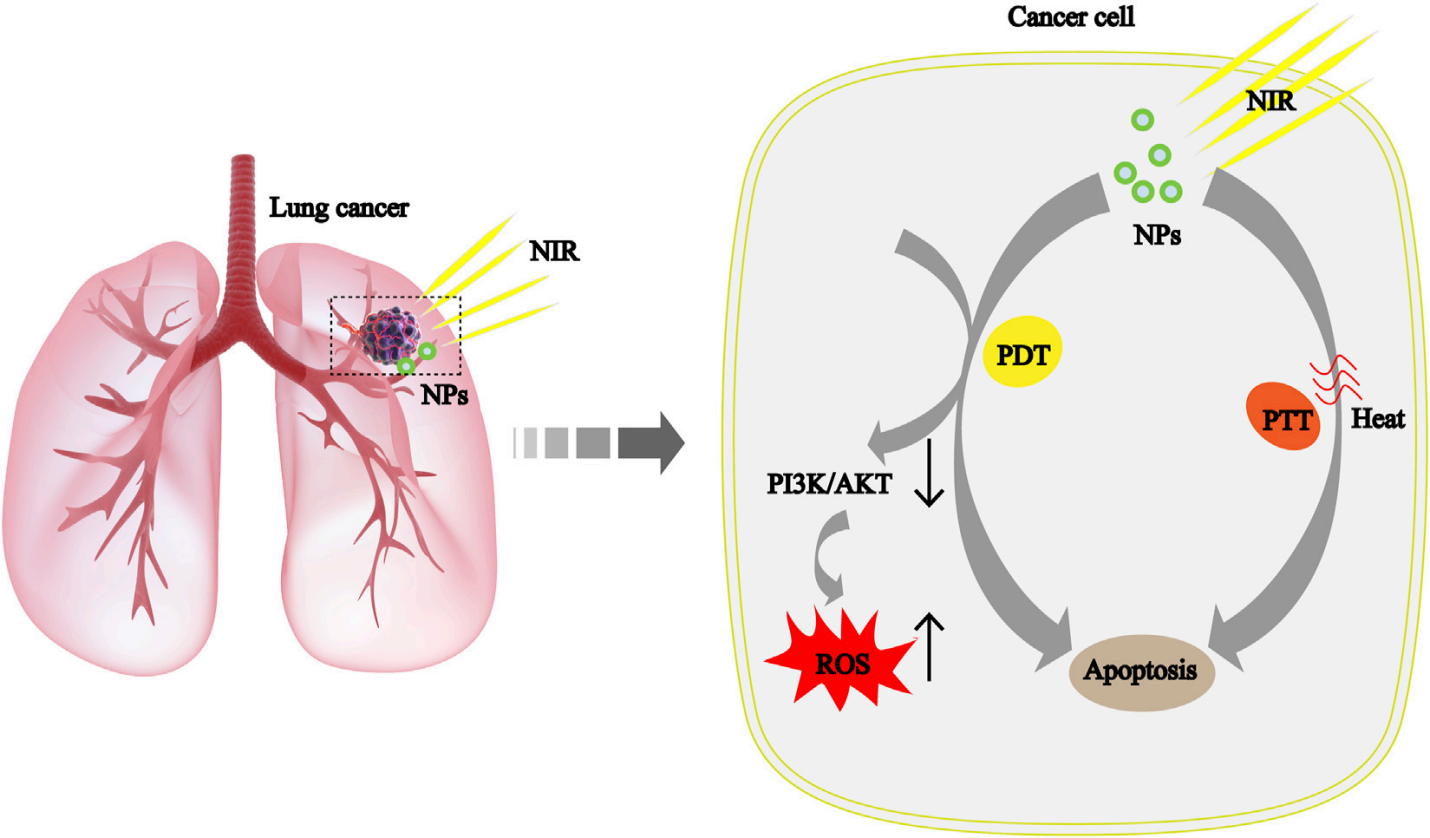
The mechanism of cancer cell apoptosis caused by the syergistic treatment of PTT and PDT based on NPs is explained scientifically in NSCLC. ROS, reactive oxygen species; NIR, near-infrared light.

In recent years, photothermal therapy (PTT) and photodynamic therapy (PDT) based on nanomaterials have made remarkable progress as an anticancer strategy, as shown in Figure 4 (Hou et al., 2020; Sun et al., 2020). Quantum dots (QDs) have good biocompatibility, solubility, excellent photostability, and easy surface functionalization properties, making them new promising nanomaterials (McHugh et al., 2018; Fan et al., 2019). Kuo et al. improved the efficiency of PDT by functionalizing nitrogen doped QDs with amino molecules (Kuo et al., 2018). Liu’s team has constructed a novel CoFe_2_O_4_ with excellent synergistic PTT/PDT properties, which can effectively inhibit NSCLC without significant toxicity. In addition, CoFe_2_O_4_ treatment also increases reactive oxygen species by regulating the PI3K/AKT pathway, thereby triggering cell death (Liu J. et al., 2021). Based on the current results, the safe, non-toxic NPs may have a positive effect on NSCLC treatment.

## 6 Nanodrug regulated JAK2/STAT3 signaling pathway in NSCLC

Janus kinase (JAK) is a non-receptor tyrosine kinase that mediates the cascade activation of signaling molecules after cytokine and receptor binding (Johnson et al., 2018). The JAK family consists of four members (JAK1-3 and TYK2) (Lin et al., 2018). Abnormal JAK2 signaling plays an important role in solid tumors (Lee et al., 2006). The signal transducer and activator of transcription (STAT) protein family consists of seven members (STAT1-4, STAT5a, STAT5b, and STAT6) (Fang et al., 2017). STAT3 is a transcription factor that has been extensively studied in cancer. Generally, the JAK2/STAT3 pathway mediates signal transduction through a variety of cytokine receptors, such as interleukin-6 (IL-6) (Zhong et al., 1994) or granulocyte colony-stimulating factor (G-CSF) (Thorn et al., 2016), and EGFR (Shi and Kehrl, 2004), which makes JAK2 phosphorylate on its tyrosine residues (called autophosphorylation) and activate its kinase domain. This signaling pathway regulates not only different cancer cell biological habits, including oncogenesis, proliferation, and distant transfer, but also the development and maintenance of tumor stem cells (CSCs). Phosphorylation levels of STAT3 are associated with poor prognosis in NSCLC, and aberrant STAT3 activity has been observed in malignant cells of other tumors. Therefore, there is a broad prospect of the STAT3 signaling pathway in the treatment of NSCLC. NPs are up-and-coming delivery systems for small-molecule drugs and nucleic acid. Consequently, the nanodrugs targeted signaling pathways are more conducive to uptake and utilization.

The TME contains a vast array of TAMs, which are classified into M1 (anti-tumor) and M2 (tumor-promoting) phenotypes (Mills, 2012; Sica and Mantovani, 2012; Komohara et al., 2016). The activation of STAT3 enhances M2-type polarization, and contributes to the formation of tumor-related phenotypes (Kortylewski et al., 2009). Chen et al. developed dual-targeting delivery system by using siRNA to target both tumor cells and M2 macrophages to silence STAT3 (Chen et al., 2020). The dual targeting system used for siRNA packaging was constructed through electronic self-assembly, consisted of carboxymethyl chitosan, and coupled with folic acid. Compared with non-targeted NPs, the dual-targeted delivery system led to a significant decrease in expression of STAT3, with a successful transition of macrophages from the M2 phenotype to the M1 phenotype, while increased apoptosis and inhibited proliferation of LC cells. In addition, Shobaki’s group used a lipid NPs (LNPs) to targeted deliver siRNA to TAMs, which can silence STAT3 and hypoxia-inducible factor 1α (HIF-1α) (Shobaki et al., 2020). It led to an increase of M1 macrophages, thus obtaining well anti-tumor therapeutic effects. On the whole, the novel nanodrugs targeting macrophages and STAT3 behave with good clinical and pharmaceutical application prospects.

RNAi is a powerful tool to downregulate the level of STAT3, which can make drug-resistant cancer cells re-sensitive (Alshamsan et al., 2011; Kotmakçı et al., 2017). To deliver RNAi-mediated plasmid DNA, Kotmakç et al. developed and evaluated a kind of cationic solid lipid NPs (SLNs). It showed that the expression level of STAT3 mRNA decreased by approximately five-fold while cisplatin-resistant cancer cells were re-sensitive after SLNs treatment (Kotmakçı et al., 2017). Chen et al. used the PEG-PLA NPs to deliver small molecule drugs (Chen et al., 2018). The results showed a synergistic anti-tumor effect on resistant NSCLC *in vitro*. The nanodrug significantly downregulated the level of biomolecules in signaling pathways, such as p-JAK2, which can cause drug resistance. In summary, the NPs-mediated drug co-delivery method can overcome drug resistance by regulating specific signaling pathways.

## 7 Nanodrug induced p53 and Bax/Bcl-2 pathway Dysfunction in NSCLC

As a well-known tumor suppressor, p53 can inhibit cell proliferation and promote apoptosis (Cui and Guo, 2016). Dysfunction of the p53 pathway is particularly common, accounting for approximately 68% of NSCLC patients (Kong et al., 2019). A variety of downstream signal molecules are regulated by p53 in tumor, such as B-cell lymphoma 2 protein (Bcl-2) and Bcl-2 related X protein (Bclx). P53 can downregulate the anti-apoptosis factor Bcl-2 and upregulate the pro-apoptosis factor Bclx (Rasheduzzaman et al., 2018).

The main problems with the application of Bcl-2 antagonists in cancer treatment are their poor water solubility and toxicity to normal cells (Schimmer et al., 2008; Paik et al., 2010). NPs can be used to improve the solubility of drugs to enhance anti-tumor efficiency (Li et al., 2018). Chen et al. prepared RBCm-OM/PLGA NPs by combining PLGA with red blood cell membrane (RBCm) to wrap obatok mesylate (OM) (Chen S et al., 2020). The results showed that their NDDS could effectively stop the growth of malignant tumor cells *in vitro* and *in vivo* by inducing apoptosis related to the high accumulation of nanodrugs. Similarly, tetrandrine (Tet) was used to make NPs (Tet NPs), which can enhance cell apoptosis by down-regulating the expression of Bcl-2. What’s more, Tet NPs can inhibit cell migration and invasion more effectively than free Tet by down-regulating matrix metalloproteinases 2 (MMP2) and matrix metalloproteinases 9 (MMP9), and upregulating tissue inhibitor of metalloproteinase-3 (TIMP-3) (Xu et al., 2014). The above results indicate that uniting anti-Bcl-2 drugs and NPs might have good prospects in the field of NSCLC treatment by improving the anti-cancer efficiency.

## 8 Nanodrug remodeled tumor immune microenvironment in NSCLC

During the process of cell differentiation and proliferation, the immunogenicity of tumor cells is reduced, which leads to immune escape (Guo et al., 2022). ICIs can influence the activation and depletion of T cells by interacting with PD-1 or PD-L1, and ultimately inhibit the occurrence of tumor immune escape. (Gordon et al., 2017). This kind of drugs have the potential to improve the survival rate of cancer patients, which are regarded as a desirable choice for the tumor treatment.

To improve the response rate of ICIs therapies, Zhao et al. successfully constructed a nanodrug consisting of fluorinated dendrimer and HSP70 promoter-driven CRISPR/Cas9 (Zhao L. et al., 2022). In this system, the activated HSP70 promoter facilitated the expression of Cas9 protein, leading to permanent genomic destruction of PD-L1. Consequently, it effectively hindered the immune escape of tumor cells. In another study, Zhou et al. found that integrin β3 (β3-INT) is highly expressed in NSCLC and further observed that the inhibitor RGDyK facilitates the process of PD-L1 ubiquitination (Zhou et al., 2022). Based on this, their group prepared RGDyK-modified mesoporous silica NPs loaded with zinc protoporphyrin (ZnPP@MSN-RGDyK). This nanodrug showed high photodynamic treatment efficiency and good immunotherapeutic effect by precisely targeting β3-INT to weaken the function of PD-L1 in preclinical tumor models.

NSCLC patients often develop drug resistance in ICIs monotherapy (Dempke et al., 2018). Kim’s team constructed an investigational tumor-targeted nanodrug named SGT-53 (Kim et al., 2022). They found that SGT-53 can restore an effective immune response to tumor cells by modulating immunosuppressive cells, including T cells and macrophages, and downregulate the expression of immunosuppressive molecule Galectin-1. Furthermore, the study revealed that the intensity of macrophage infiltration was highly correlated with the emergence of ICIs resistance (Cui et al., 2020; Kim et al., 2020; Zhu et al., 2020). Besides, Xu et al. bound nano-diamond doxorubicin conjugate (Nano-DOX) to a PD-L1 blocking agent named BMS-1, which can effectively reactivate the M1-type macrophages to kill tumor cells and inhibit tumor growth (Xu et al., 2021).

In summary, the combination of NPs and ICIs can not only help in the precise targeting of drugs, but also restore the immune surveillance function.

## 9 Challenges and prospects of nanodrug in targeted NSCLC therapy

Targeted therapies and immunotherapies have been widely used in clinical practice. These drugs improve the prognosis of patients with a variety of advanced cancers. However, most of the targeted drugs are unable to achieve the expected therapeutic effects due to their low solubility, low bioavailability and severe adverse reactions (de Scordilli et al., 2022). Furthermore, systemic administration of immunotherapy can also cause immune damage to respiratory and cardiovascular systems or other systems, and can even be life-threatening (Lahiri et al., 2023). Nanotechnology is a rapidly evolving field that offers new strategies related to drug delivery. Here, the modifiability and micro-size properties enable nanomatrials to play a synergistic therapeutic role with drugs. From this, NDDS can regulate the drug concentration of target cells by controlling the release rate, which eventually overcome the shortcomings of anti-cancer drugs, including the drug resistance, systemic toxicity and rapid metabolism (Abdelaziz et al., 2018; Wang X. et al., 2021). The intersection of disciplines brings a broader prospect for the development of nanomedicine.

Despite the encouraging successes, there are still many problems that remain to be solved, which seem impossible to accomplish in a short period of time. Currently, many studies are dedicated to develop safe and effective nanodrugs in NSCLC, but very few drugs can pass clinical trials (Liao et al., 2020). There are still many unknown metabolic pathways of nanodrugs in the human body, which may bring unpredictable drug side effects (Abdelaziz et al., 2018). They are easily absorbed by healthy cells through active transport because the nanodrugs have small size, which may cause damage on normal histiocytes (Ferrari, 2005; Wang X. et al., 2021). There is still a long way to go for nanodrugs to enter clinical treatment, which requires close cooperation between disciplines. For example, multi-radionuclide imaging can personalize treatment by stratifying patients, and the use of artificial intelligence algorithms can help select specific nano parameters in these highly complex cases to improve the biological function (Arrieta et al., 2012; Mahmoud et al., 2020). Nanodrug plays an active role in the regulation of various signaling pathways in tumors. Many signaling pathways in TME have been shown to play important roles in tumor cell growth, and it is necessary to develop specific drug and delivery devices for these signaling pathways (Wang F. et al., 2021; Lei et al., 2022).

In summary, both nanotechnology and signaling pathway-regulated drugs are ushering in a new era of disease treatment, and their synergistic effects have broad clinical application prospects. However, there are still many problems need to be solved. Accordingly, it is necessary to combine the advantages of complementary multidisciplinary to solve these problems, and this will bring benefits to tumor patients as soon as possible.

## 10 Conclusion and future prospects

In recent years, the global incidence of NSCLC is the highest among all cancer incidence rates. Currently, precision molecular therapy for NSCLC has been widely used, and many drugs have entered the clinic, but more signal-regulated pathways of tumor development are still unclear. It is the goal of medical development to find more effective and safer therapeutic targets. NDDS brings more convenience to drug therapy, but the defects of the nanodrug itself still need to be overcome. The most important problems come from the stability, effectiveness, and safety of the nanomaterials themselves, in addition to the type of carrier material, preparation technology, and cost issues (Ji et al., 2022). At present, the pharmacokinetic behaviors of more nanodrugs are unclear, which is also an important reason for the low success rate of clinical transformation (Cao and Chen, 2022). Multidisciplinary intersection brings the advantage of solving these problems, and therefore the exchange of new technologies among various disciplines needs to be enhanced (Boehnke et al., 2022; Xu C. et al., 2023). Besides, studies on the regulation of signaling pathways by nanodrugs are mostly conducted *in vitro* or in animal models. Due to the heterogeneity of cancers in animal models and clinical patients, the therapeutic effects of nanodrugs have large differences between the preclinical therapy and clinical trials, which reduces their clinical application (Xu W. et al., 2023). Therefore, it will be beneficial to improve clinical feasibility of nanodrugs by developing humanized animal models (May, 2018). Meanwhile, we need clinical trials to validate and evaluate their efficacy and safety.

The combination of drugs and nanomaterials will provide hope for patient survival (Van der Meel et al., 2019; Jia et al., 2023). Multi-drug combination is one of the important means to improve anti-cancer efficacy, and it is also a general trend in the field of drug research and development (Detappe et al., 2023). Exploring more therapeutic targets for molecular signaling is also an important prerequisite for promoting multi-drug combination therapy (You et al., 2023). Nanocarriers provide a safe, fast and effective platform for multi-drug combination therapy (Yang et al., 2023). Hence, NDDS combined with multi-target drugs synergistic therapy may be the focus of future research.

Designing efficient and safe nanodrugs and exhaustively investigating their pharmacokinetics *in vivo* are key to their application and development. The development of humanised animal models will also greatly improve the success rate of clinical translation of novel nanodrugs. Exploring more molecular signalling targets and multi-target combination therapy based on nanocarriers are important research directions. With the development of nanotechnology, NPs will also be updated, and their obvious advantages as drug carriers will be played more in practical applications. Eventually, they will become powerful tools for patients to overcome NSCLC. For the next phase of research on nanodrug, researchers should spend more time and effort to provide clear evidence for existing mechanisms rather than creating many new and complex nanocarriers for similar concepts as a way to advance the clinical translation of nanomedicines.

In conclusion, the research of nanodrugs in regulating signaling pathways is still in its infancy, and many practical problems still need to be solved.
